# Telomere Length, *TERT*, and miRNA Expression

**DOI:** 10.1371/journal.pone.0162077

**Published:** 2016-09-14

**Authors:** Martha L. Slattery, Jennifer S. Herrick, Andrew J. Pellatt, Roger K. Wolff, Lila E. Mullany

**Affiliations:** Department of Medicine, University of Utah, 383 Colorow, Salt Lake City, Utah 84108, United States of America; National Institute of Environmental Health Sciences, UNITED STATES

## Abstract

It has been proposed that miRNAs are involved in the control of telomeres. We test that hypothesis by examining the association between miRNAs and telomere length (TL). Additionally, we evaluate if genetic variation in telomerase reverse transcriptase (*TERT*) is associated with miRNA expression levels. We use data from a population-based study of colorectal cancer (CRC), where we have previously shown associations between TL and *TERT* and CRC, to test associations between TL and miRNA expression and TERT and miRNA expression. To gain insight into functions of miRNAs associated with *TERT* we tested linear associations between miRNAs and their targeted gene mRNAs. An Agilent platform that contained information on over 2000 miRNAs was used. TL was measured using a multiplexed quantitative PCR (qPCR). RNAseq was used to assess gene expression. Our sample consisted of 1152 individuals with SNP data and miRNA expression data; 363 individuals with both TL and miRNA; and 148 individuals with miRNA and mRNA data. Thirty-three miRNAs were directly associated with TL after adjusting for age and sex (false discovery rate (FDR) of 0.05). *TERT* rs2736118 was associated with differences in miRNA expression between carcinoma and normal colonic mucosa for 75 miRNAs (FDR <0.05). Genes regulated by these miRNAs, as indicated by mRNA/miRNA associations, were associated with major signaling pathways beyond their TL-related functions, including PTEN, and PI3K/AKT signaling. Our data support a direct association between miRNAs and TL; differences in miRNA expression levels by *TERT* genotype were observed. Based on miRNA and targeted mRNA associations our data suggest that *TERT* is involved in non-TL-related functions by acting through altered miRNA expression.

## Introduction

MicroRNAs (miRNA) are non-coding RNAs that function as gene regulators [1]. As such, they provide a mechanism whereby genes involved in various signaling pathways can be regulated simultaneously [2]. Both aging and cellular senescence are thought to be regulated in part by miRNAs [3, 4]. It also has been suggested that miRNAs control the function of telomeres in cancer [5]. Telomeres, located at the end of chromosomes, shorten with every cell division; when they become too short the cell is unable to divide, leading to the induction of senescence and apoptosis, which are key features of normal cell function, but absent in tumor cells. Telomere length (TL) has been associated with colorectal cancer, with higher risk of disease for both long and short telomeres [6, 7]. Telomerase, a ribonucleoprotein enzyme that is essential for the replication of chromosome ends, is low or non-expressing in normal human somatic cells, but has been shown to be expressed in the majority of tumor cells [8]. Telomerase reverse transcriptase (TERT) is essential for maintenance of telomere length by protecting chromosome ends. TERT has been proposed to regulate miRNAs by regulation of miRNA biogenesis, with diminished primary miRNA expression in TERT-reduced cells [9], and has been shown to be associated with both TL and cancer [6, 10–14]. TERT is thought to be one of the most critical, if not the rate-limiting step, in production of telomerase activity [8, 15]. We have shown previously that *TERT* rs2853676 was associated with TL and that TL was associated with colorectal cancer [11]. Additionally *TERT*-CLPTM1L rs2853668 and *TERT* rs2736118 were associated with colorectal cancer risk in this study population [14].

In this paper we examine the associations between TL and miRNA expression in colorectal tissue. Additionally we test the hypothesis that variation in TERT influences miRNA expression levels. We focus on associations between miRNAs and *TERT* SNPs previously associated with colorectal cancer and examine miRNA expression in normal colonic mucosa tissue as well as the differential expression of miRNAs between carcinoma and normal colonic mucosa tissue.

## Materials and Methods

### Study population

The study population consisted of individuals previously enrolled in a study of Diet, Lifestyle, and Colon cancer at the University of Utah and the Kaiser Permanente Medical Research Program (KPMRP) [16] for whom both miRNA from carcinoma and adjacent normal colonic mucosa tissue were available. Study subjects included incident cases of colon cancer between the ages of 30 and 79 who were non-Hispanic white, Hispanic, or African American, and were able to provide a signed informed consent prior to participation in the study. The study was approved by the University of Utah Institutional Review Board for Human Subjects.

### miRNA processing

RNA (miRNA) was extracted from formalin-fixed paraffin embedded tissues. We assessed slides and tumor blocks that were prepared over the duration of the study prior to the time of miRNA isolation to determine their suitability. The study pathologist reviewed slides to delineate carcinoma and normal colonic mucosa tissue. Cells were dissected from 1–4 sequential sections on aniline blue stained slides using an H&E slide for reference. Total RNA containing miRNA was extracted, isolated, and purified using the RecoverAll Total Nucleic Acid isolation kit (Ambion); RNA yields were determined using a NanoDrop spectrophotometer. 100 ng total RNA was labeled with Cy3 and hybridized to Agilent Human miRNA Microarray V19.0 and were scanned on an Agilent SureScan microarray scanner model G2600D. The Agilent Human microarray was generated using known miRNA sequence information compiled in the Sanger miRBase database v19.0. The microarray contains probes for 2006 unique human miRNAs. The miRNA array contains 60,000 unique human sequences and averages 30 replicates per probe sequence. Data were extracted from the scanned image using Agilent Feature Extract software v.11.5.1.1. Data were required to pass stringent QC parameters established by Agilent that included tests for excessive background fluorescence, excessive variation among probe sequence replicates on the array, and measures of the total gene signal on the array to assess low signal. If samples failed to meet quality standards for any of these parameters, the sample was re-labeled, hybridized to arrays, and scanned. If a sample failed QC assessment a second time the sample was deemed to be of poor quality and the individual was excluded from down-stream analysis. A 75^th^ percentile scaling was performed to normalize across arrays was done using preprocessCore [17] (www.bioconductor.org) to minimize differences that could be attributed to the array, amount of RNA, location on array, or other factors that could erroneously influence expression measurements. Testing of both reliability and comparative validity were done [18]. We showed a 98% repeatability in the Agilent platform between repeat samples. Additionally, the Agilent platform had fairly good agreement with Nanostring results and 100% agreement in expression values and fold change with qPCR results [19].

### RNA-Seq Sequencing Library Preparation and Data Processing

Total RNA was available from 197 carcinoma and normal mucosa pairs. These samples were taken from the study subjects used for miRNA analysis and were extracted, isolated and purified in the same manner as previously described [20]. RNA library construction was done with the Illumina TruSeq Stranded Total RNA Sample Preparation Kit with Ribo-Zero. The samples were then fragmented and primed for cDNA synthesis, adapters were then ligated onto the cDNA, and the resulting samples were then amplified using PCR; the amplified library was then purified using Agencount AMPure XP beads. A more detailed description of the methods can be found in our previous work [21].

Sequencing was done using an Illumina TruSeq v3 single read flow cell and a 50 cycle single-read sequence run was performed on an Illumina HiSeq instrument. Reads were then aligned to a sequence database containing the human genome (build GRCh37/hg19, February 2009 from genome.ucsc.edu) and alignment was performed using novoalign v2.08.01. Python and a pysam library were used to calculate counts for each exon and UTR of the genes using a list of gene coordinates obtained from http://genome.ucsc.edu. We dropped features that were not expressed in our data or for which the expression was missing for the majority of samples. A more detailed description of the methods can be found in our previous work [21].

### Telomere Length (TL) Telomere-related Gene Analysis

TL was measured using a multiplexed quantitative PCR (qPCR) method previously described by Cawthon [22]. This method modified earlier qPCR methods in which telomere signals were measured separately from single copy gene signals in order to produce a T/S ratio corresponding to TL, which is proportional to the average telomere length in a cell. Therefore a larger T/S ratio represents a longer telomere length. The multiplexed PCR analysis uses a single dye and measures both the telomere signals and single copy gene signals in the same well. This is achieved by using CG clamps to stabilize the single copy gene giving it a higher melting point. The unique sequence is then amplified during a different cycle than the telomeric sequence, allowing for a single qPCR to determine the T/S ratio [6].

DNA was obtained from immortalized cell lines from study participants for the colon cancer study and from whole blood for the rectal cancer study. To assure the quality of telomere assay for these samples from different DNA origins we evaluated associations with TL separately for cases and controls from each study and as shown previous, results were similar for both study groups and we concluded that DNA source did not significantly alter TL length in this study. Two samples yielded a T/S ratio greater than three and were excluded. These samples were excluded because we concluded, through gel electrophoresis, that the DNA was degraded and therefore the T/S ratio was unreliable. We focused on Single Nucleotide Polymorphisms (SNPs) that were previously associated with TL and/or colon cancer [6, 14]. We analyzed *TERT*-*CLPTM1L* rs2853668 which is 5.1kb upstream of *TERT* and has been associated with colon cancer in GWAS [23]. We also test *TERT* rs2736118 and *TERT* rs2853676 with miRNA expression.

### Statistical Analysis

Our sample consisted of 1152 individuals with both SNP data and miRNA expression data; 363 individuals with both TL and miRNA expression data in the normal colonic mucosa; and 148 individuals who had both miRNA and mRNA data, and 176 cases in the colon cancer study and 184 cases in the rectal cancer study had both TL and SNP data.

Three major components of the analysis were conducted. First, we evaluated the linear associations between TL and those miRNAs for which at least 20% of the population had expression (N = 817 miRNA) by fitting a linear regression model to the log base 2 transformed expression levels; we adjusted for age and sex. P-values for each regression analysis were generated from the bootstrap method by creating a distribution of 1,000 β coefficients for each miRNA and evaluating H_0_: β = 0 vs. H_1_: β≠0 using the boot package in R. We adjusted for multiple comparisons using a false discovery rate (FDR) level of 0.05 [24]. We standardized the slopes generated from the overall dataset in order to compare the results across the miRNA.

Second, we assigned inheritance models for *TERT* SNPs based on previous findings for colorectal cancer risk using either dominant or recessive models [14]. We compared log base 2 transformed expression levels across the selected genotype models using the significance analysis of microarrays (SAM) technique implemented in the R package siggenes; p-values were based on 1,000 permutations. We utilized an FDR level of 0.05 to determine which *TERT* SNP/miRNA associations were significant after adjustment for multiple comparisons.

For both the TL/miRNA analysis and the TERT SNP/miRNA analysis, we evaluated miRNA expression in both normal colonic mucosa and for differentially expressed miRNAs (i.e. difference between carcinoma tissue and normal colonic mucosa). Looking at normal colonic mucosa allowed us to evaluate the effect of TL or SNP on overall miRNA expression. However, since an association with normal colonic mucosa could influence baseline level of the miRNA expressed and could potentially miss important changes in miRNA expression between carcinoma and normal colonic mucosa, we also evaluated miRNA differential expression. This allowed us to gain insight into regulation of miRNAs that could be more directly associated with cancer through their influence on carcinoma miRNA expression; by comparing to normal expression we were able to control for differences in tumor subsite.

One of the goals of the study was to identify non-telomere related mechanisms whereby *TERT* may be operating through altered miRNA expression levels. To accomplish this, we undertook the third part of the analysis, where we used miRTarBase (www.http://mirtarbase.mbc.nctu.edu.tw/) v6.0 (as of 09/15/2015) [25] to identify validated target genes for the significant miRNAs identified in our *TERT* SNP/miRNA analysis. We calculated the differential mRNA expression as the difference of the log base 2 of the RPKM (Reads per Kilobase per Million) for the carcinoma and normal mucosa tissues for the 4047 target genes identified by miRTarBase. We then evaluated the 6309 combinations of miRNAs with their respective mRNA target genes. We used the bootstrap method as described above to assess any linear relationship between the differential mRNA and miRNA expression levels, adjusting for age and sex.

QIAGEN’s Ingenuity Pathway Analysis (IPA) (QIAGEN Redwood City, www.qiagen.com/ingenuity) [26] was then used to evaluate the genes that were dysregulated in conjunction with the miRNAs. We considered direct relationships from the IPA knowledge base of genes only, limited to experimentally verified and mammalian results only, and considered all mutations, data sources, and tissues. We report canonical pathways that were significant enriched with these genes after adjustment for multiple comparisons.

## Results

The average age of study participants from the colon cancer study was slightly older than from the rectal cancer study (Table 1). The distribution of age, sex, and AJCC stage was similar for those individuals included in the smaller TL dataset as with the larger SNP dataset.

**Table 1:** Description of study population (includes cases only).

		Colon		Rectal	
		Mean	SD	Mean	SD
Telomere Length (TL)^1^(T/S ratio)		1.17	0.55	1.20	0.29
Age (years)		65.3	9.1	61.9	10.9
		N	%	N	%
Sex	Male	380	55.7	280	59.6
	Female	302	44.3	190	40.4
AJCC Stage	1	167	24.7	213	45.8
	2	220	32.6	88	18.9
	3	222	32.9	136	29.2
	4	66	9.8	28	6.0
*TERT* (rs2736118)^2^	AA	341	51.5	237	53.1
	AG/GG	321	48.5	209	46.9
*TERT* (rs2853668)	GG/GT	631	93.3	445	94.7
	TT	45	6.7	25	5.3
*TERT* (rs2853676)	CC	348	53.0	155	58.5
	CT/TT	309	47.0	110	41.5

^1^ 179 individuals in the colon cancer study and 184 individuals in the rectal cancer study had TL, miRNA and SNP data.

^2^ 53 individuals with AA genotype had RNAseq data; 50 individuals with the AG/GG genotype had RNAseq; 148 individuals had both miRNA and mRNA (RNAseq) data.

Assessment of the linear relationship between TL and miRNA expression levels in for the 363 individuals with both TL and miRNA from normal colorectal mucosa showed that 34 miRNAs were associated with TL after adjustment for age and sex (Table 2). Thirty-three of the miRNAs were directly associated with TL, in that TL increased as miRNA expression level increased. Only miR-487b was inversely associated with TL, showing lower levels of miR-487b expression with longer TL length.

**Table 2:** Associations between TL (T/S Ratio) and miRNA expression levels adjusted for age (FDR<0.05).

miRNA	Mean Expression	Beta Coefficient^1^	P-value^2^	Q-Value
hsa-miR-1185-2-3p	6.81	0.17	< .0001	< .0001
hsa-miR-1207-5p	10.65	0.14	0.002	0.0481
hsa-miR-1226-5p	5.5	0.16	0.002	0.0481
hsa-miR-1229-5p	9.07	0.14	0.002	0.0481
hsa-miR-1247-3p	4.64	0.12	0.002	0.0481
hsa-miR-134	7.84	0.16	< .0001	< .0001
hsa-miR-3141	7.4	0.14	0.002	0.0481
hsa-miR-3158-5p	4.82	0.16	< .0001	< .0001
hsa-miR-3162-5p	11.19	0.15	0.002	0.0481
hsa-miR-3194-5p	7	0.14	0.002	0.0481
hsa-miR-3196	10.19	0.18	< .0001	< .0001
hsa-miR-3200-5p	4.75	0.17	0.002	0.0481
hsa-miR-3937	5.8	0.14	0.002	0.0481
hsa-miR-4253	5.29	0.18	< .0001	< .0001
hsa-miR-4459	14.38	0.15	< .0001	< .0001
hsa-miR-4496	5.74	0.17	< .0001	< .0001
hsa-miR-4497	11.57	0.17	0.002	0.0481
hsa-miR-4530	12.75	0.15	< .0001	< .0001
hsa-miR-4535	5.93	0.15	< .0001	< .0001
hsa-miR-4673	6.3	0.16	0.002	0.0481
hsa-miR-4689	6.64	0.16	0.002	0.0481
hsa-miR-4739	9.71	0.18	< .0001	< .0001
hsa-miR-487b	1.6	-0.17	< .0001	< .0001
hsa-miR-5006-5p	9.36	0.15	< .0001	< .0001
hsa-miR-5088	5.64	0.15	0.002	0.0481
hsa-miR-5195-3p	7.14	0.14	0.002	0.0481
hsa-miR-575	9.18	0.15	< .0001	< .0001
hsa-miR-601	5.31	0.15	< .0001	< .0001
hsa-miR-6076	7.84	0.14	0.002	0.0481
hsa-miR-662	5.32	0.16	0.002	0.0481
hsa-miR-718	6.59	0.18	< .0001	< .0001
hsa-miR-762	10.72	0.15	0.002	0.0481
hsa-miR-887	5.74	0.2	< .0001	< .0001
hsa-miR-939-5p	9.06	0.14	< .0001	< .0001

^1^Beta coefficients are from a linear regression of TL on miRNA expression after adjustment for age, sex, and study

Evaluation of differences in miRNA expression levels between carcinoma tissue and normal colorectal mucosa showed that *TERT* rs2736118 was associated with differential miRNA expression based on genotype (Table 3). *TERT* rs2736118 was associated with 75 miRNAs with an FDR of <0.05. Individuals with the AA (homozygous dominant) genotype were more likely to have greater miRNA expression in the tumor than in the normal colorectal mucosa (61% of the time) while individuals with the AG/GG genotypes were more likely to have greater miRNA expression in the normal colorectal mucosa (84% of the time). Neither *TERT-CLPTMIL* rs2853668 nor *TERT* rs2853676 significantly altered miRNA expression in normal colonic mucosa or miRNA expression between carcinoma and normal colorectal mucosa.

**Table 3:** Associations between *TERT* rs2736118 and miRNA differential expression^1^ with an FDR of <0.05.

	AA (N = 556)			AG/GG (N = 508)			
miRNA	Mean Tumor Expression	Mean Normal Expression	Mean Difference in Expression (T-N)	Mean Tumor Expression	Mean Normal Expression	Mean Difference in Expression (T-N)	Q-values
hsa-miR-1203	0.9040	1.2164	-0.3124	0.7861	1.3040	-0.5179	0.0044
hsa-miR-1237-5p	2.7048	2.8009	-0.0961	2.5824	2.8954	-0.313	0.0003
hsa-miR-125b-1-3p	3.3566	3.3173	0.0393	3.2864	3.3280	-0.0416	0.0069
hsa-miR-1266	0.9616	0.7167	0.2449	0.8296	0.7950	0.0345	0.0061
hsa-miR-1276	1.1104	1.0311	0.0793	0.9951	1.1146	-0.1195	0.0083
hsa-miR-1295b-3p	1.0382	1.0006	0.0376	0.9074	1.0876	-0.1803	0.0055
hsa-miR-1323	2.2143	2.1357	0.0786	2.0477	2.2264	-0.1788	0.0011
hsa-miR-1470	2.0574	2.3573	-0.2999	1.8649	2.4017	-0.5368	0.0008
hsa-miR-184	0.9277	0.9309	-0.0032	0.7507	1.0149	-0.2641	0.0011
hsa-miR-206	2.8032	3.1652	-0.3621	2.6350	3.2131	-0.5781	0.0109
hsa-miR-302c-5p	1.2712	0.9455	0.3257	1.1561	1.0997	0.0564	0.0001
hsa-miR-3122	0.8753	0.8425	0.0328	0.7107	0.8545	-0.1437	0.0151
hsa-miR-3131	4.0127	3.8855	0.1272	3.9049	3.9703	-0.0654	0.0001
hsa-miR-3150b-3p	1.0041	0.8180	0.1861	0.8778	0.8914	-0.0136	0.0100
hsa-miR-3161	3.8361	3.9214	-0.0852	3.8044	3.9553	-0.1509	0.0080
hsa-miR-3177-5p	1.9296	2.0896	-0.16	1.7230	2.1565	-0.4335	0.0003
hsa-miR-3181	0.7395	1.0470	-0.3075	0.6513	1.1449	-0.4936	0.0155
hsa-miR-3186-3p	0.7814	0.6846	0.0968	0.6631	0.7670	-0.1039	0.0046
hsa-miR-3189-5p	2.1073	2.1018	0.0055	2.0026	2.2128	-0.2102	0.0087
hsa-miR-339-3p	1.9875	1.8349	0.1527	1.8464	1.8830	-0.0366	0.0050
hsa-miR-34c-3p	2.3869	2.3467	0.0401	2.3206	2.4220	-0.1014	0.0152
hsa-miR-3615	0.7203	0.9008	-0.1804	0.6091	0.9742	-0.3651	0.0071
hsa-miR-3660	1.9127	1.9104	0.0022	1.7900	1.9813	-0.1913	0.0158
hsa-miR-3679-3p	0.4062	0.3906	0.0156	0.2972	0.4282	-0.131	0.0011
hsa-miR-3680-3p	2.9284	2.6281	0.3003	2.7893	2.6970	0.0923	0.0008
hsa-miR-378e	1.0146	0.8963	0.1183	0.8816	0.9639	-0.0824	0.0037
hsa-miR-3922-5p	0.6227	0.4820	0.1406	0.5307	0.5691	-0.0384	0.0031
hsa-miR-4300	0.8662	0.7173	0.1488	0.7436	0.7864	-0.0428	0.0022
hsa-miR-4303	0.8538	0.9330	-0.0792	0.7200	0.9955	-0.2755	0.0138
hsa-miR-4421	0.5460	0.7538	-0.2078	0.4893	0.8849	-0.3956	0.0113
hsa-miR-4436a	2.0374	1.9462	0.0911	1.9109	2.0796	-0.1688	0.0039
hsa-miR-4444	3.2323	2.9691	0.2632	3.0676	3.0396	0.028	0.0011
hsa-miR-4450	1.8216	1.6023	0.2192	1.7353	1.7504	-0.0151	0.0072
hsa-miR-4461	1.9050	1.7542	0.1507	1.7372	1.8575	-0.1203	0.0014
hsa-miR-4479	0.7581	0.7816	-0.0235	0.6689	0.8772	-0.2083	0.0158
hsa-miR-4489	3.5472	3.6088	-0.0616	3.4330	3.6676	-0.2347	0.0135
hsa-miR-4510	2.4625	2.5053	-0.0429	2.3292	2.5568	-0.2276	0.0064
hsa-miR-4519	3.0204	2.9033	0.1171	2.8279	2.9748	-0.1469	0.0004
hsa-miR-4522	3.4239	3.6634	-0.2395	3.2646	3.6946	-0.4300	0.0033
hsa-miR-4526	1.8260	1.8567	-0.0307	1.6271	1.8806	-0.2535	0.0013
hsa-miR-4638-5p	1.4805	1.5457	-0.0652	1.3145	1.6703	-0.3559	0.0014
hsa-miR-4654	1.3087	1.0013	0.3074	1.1610	1.1001	0.0609	0.0022
hsa-miR-4657	1.5101	1.0805	0.4295	1.3891	1.1962	0.1929	0.0032
hsa-miR-4659b-3p	1.7201	1.9028	-0.1827	1.5847	2.0117	-0.4271	0.0081
hsa-miR-4660	2.0659	1.8028	0.2631	1.9383	1.9375	0.0008	0.0043
hsa-miR-4674	2.5960	2.6555	-0.0594	2.4912	2.7185	-0.2273	0.0029
hsa-miR-4676-5p	1.0025	0.8863	0.1162	0.9258	1.0054	-0.0796	0.0127
hsa-miR-4682	0.7889	0.6533	0.1356	0.6994	0.7349	-0.0355	0.0114
hsa-miR-4684-3p	1.1204	1.1684	-0.048	1.0038	1.2391	-0.2353	0.0083
hsa-miR-4715-5p	1.6858	1.6206	0.0652	1.4839	1.7073	-0.2233	0.0007
hsa-miR-4717-3p	2.0039	1.7400	0.2639	1.7938	1.7920	0.0018	0.0001
hsa-miR-4731-3p	0.7447	0.6289	0.1158	0.6076	0.7042	-0.0966	0.0011
hsa-miR-4732-5p	4.1588	3.8034	0.3555	4.1413	3.9066	0.2347	0.0089
hsa-miR-4748	2.3375	2.2502	0.0874	2.1273	2.2557	-0.1285	0.0084
hsa-miR-500a-3p	1.5768	1.4419	0.1348	1.4121	1.5348	-0.1227	0.0006
hsa-miR-516b-5p	2.1095	2.0461	0.0634	1.9679	2.2117	-0.2438	0.0005
hsa-miR-518c-5p	0.7122	0.8026	-0.0904	0.6082	0.8813	-0.2731	0.0101
hsa-miR-5195-5p	2.8941	2.6472	0.2469	2.7678	2.7966	-0.0287	0.0020
hsa-miR-519e-5p	1.0583	1.0823	-0.024	0.9249	1.2156	-0.2907	0.0003
hsa-miR-520e	3.2490	3.6373	-0.3883	3.1534	3.7298	-0.5763	0.0068
hsa-miR-525-5p	0.8990	0.7384	0.1606	0.7517	0.8720	-0.1203	0.0000
hsa-miR-550a-5p	2.9657	3.0505	-0.0848	2.8758	3.0853	-0.2096	0.0097
hsa-miR-551b-5p	0.8574	0.6666	0.1908	0.7783	0.7348	0.0436	0.0109
hsa-miR-5572	2.7054	2.6641	0.0413	2.4570	2.6748	-0.2178	0.0010
hsa-miR-5584-5p	0.4473	0.4448	0.0024	0.3820	0.5262	-0.1442	0.0066
hsa-miR-566	1.7370	1.8000	-0.0631	1.6106	1.8912	-0.2806	0.0019
hsa-miR-583	2.7128	1.5976	1.1152	2.6492	1.7677	0.8814	0.0065
hsa-miR-616-3p	0.8733	0.5788	0.2945	0.8258	0.6369	0.1889	0.0157
hsa-miR-639	3.0031	3.1208	-0.1177	2.9192	3.1285	-0.2093	0.0072
hsa-miR-6509-5p	3.6249	3.6661	-0.0411	3.5866	3.7262	-0.1396	0.0104
hsa-miR-658	0.5168	0.6255	-0.1087	0.4250	0.6472	-0.2221	0.0055
hsa-miR-659-3p	3.4147	3.2518	0.1629	3.2092	3.3117	-0.1025	0.0003
hsa-miR-6716-5p	2.2443	2.1112	0.1331	2.1058	2.1845	-0.0787	0.0017
hsa-miR-6718-5p	2.9358	2.7755	0.1604	2.8682	2.8986	-0.0304	0.0008
hsa-miR-708-5p	3.1203	3.2337	-0.1133	3.0296	3.3207	-0.2911	0.0122

^1^Expression based on log2 transformed data

The 75 miRNAs dysregulated by *TERT* rs2736118 were associated with 4047 genes and 6309 miRNA/mRNA associations according to miRTarBase. To determine which of these genes might be associated with mRNA regulation in colonic tissue, we assessed each miRNA with its mRNA gene target in colonic carcinoma tissue and colonic normal mucosa using a linear regression. We identified 573 miRNA/mRNA associations p-value of <0.05 prior to adjustment for multiple comparisons. Of these, 150 significant miRNA/mRNA target associations remained when the FDR was 0.05 and 270 miRNA/mRNA associations remained with the FDR of <0.1 (Table 4). The miRNAs and their targeted genes shown in Table 4 also indicate the direct of the association based on the beta coefficient. Of note is that the associations were limited to 13 miRNAs. All but five of these associations showed inverse associations between the miRNA and the mRNA.

**Table 4:** Regression of differential miRNA expression and differential mRNA expression (FDR<0.05).

miRNA	Gene	Beta	FDR Q
hsa-miR-3161	*WIZ*	-0.24	0.006
hsa-miR-3161	*SLC25A40*	-0.27	0.006
hsa-miR-3161	*EFNB2*	-0.11	0.006
hsa-miR-3161	*TRIM5*	-0.28	0.006
hsa-miR-3161	*PPIG*	-0.24	0.006
hsa-miR-3161	*RELA*	-0.2	0.006
hsa-miR-3161	*CSTF2T*	-0.27	0.006
hsa-miR-3161	*SRGAP1*	-0.31	0.006
hsa-miR-3161	*HMGN2*	-0.26	0.006
hsa-miR-3161	*NUP50*	-0.23	0.009
hsa-miR-3161	*TXNDC12*	-0.25	0.009
hsa-miR-3161	*BCL2L11*	-0.2	0.009
hsa-miR-3161	*RYBP*	-0.25	0.009
hsa-miR-3161	*TMEM41A*	-0.2	0.009
hsa-miR-3161	*PRDX3*	-0.29	0.009
hsa-miR-3161	*WIPF2*	-0.23	0.009
hsa-miR-3161	*TMEM30B*	-0.31	0.009
hsa-miR-3161	*CTNND1*	-0.22	0.009
hsa-miR-3161	*VMP1*	-0.19	0.012
hsa-miR-3161	*TRIP11*	-0.26	0.012
hsa-miR-3161	*INVS*	-0.24	0.012
hsa-miR-3161	*GEMIN6*	-0.17	0.012
hsa-miR-3161	*EOGT*	-0.27	0.012
hsa-miR-3161	*UBXN2A*	-0.2	0.012
hsa-miR-3161	*NUPL2*	-0.18	0.016
hsa-miR-3161	*PCMTD1*	-0.16	0.016
hsa-miR-3161	*TOM1L2*	-0.21	0.016
hsa-miR-3161	*PTP4A1*	-0.18	0.017
hsa-miR-3161	*QKI*	-0.23	0.017
hsa-miR-3161	*SHFM1*	-0.17	0.017
hsa-miR-3161	*THRB*	-0.18	0.017
hsa-miR-3161	*SREK1*	-0.21	0.017
hsa-miR-3161	*ACTBL2*	0.15	0.017
hsa-miR-3161	*CCDC43*	-0.24	0.017
hsa-miR-3161	*PSMC2*	-0.15	0.02
hsa-miR-3161	*ARMC10*	-0.18	0.02
hsa-miR-3161	*SPOP*	-0.27	0.023
hsa-miR-3161	*HIF1AN*	-0.22	0.023
hsa-miR-3161	*ZNF706*	-0.14	0.026
hsa-miR-3161	*ZNF100*	-0.21	0.028
hsa-miR-3161	*MCTS1*	-0.17	0.031
hsa-miR-3161	*ZNF431*	-0.17	0.034
hsa-miR-3161	*ABHD2*	-0.07	0.036
hsa-miR-3161	*PDIK1L*	-0.21	0.036
hsa-miR-3161	*ELOVL5*	-0.12	0.041
hsa-miR-3161	*BTC*	-0.22	0.041
hsa-miR-3161	*NUP37*	-0.09	0.045
hsa-miR-3161	*TPCN2*	-0.19	0.045
hsa-miR-3161	*LRRC40*	-0.25	0.048
hsa-miR-3161	*DSE*	-0.19	0.052
hsa-miR-3161	*PPIL1*	-0.14	0.055
hsa-miR-3161	*VSNL1*	-0.19	0.057
hsa-miR-3161	*STARD7*	-0.33	< .001
hsa-miR-3161	*SRP54*	-0.3	< .001
hsa-miR-3161	*RBM3*	-0.22	< .001
hsa-miR-3161	*DGKH*	-0.15	< .001
hsa-miR-3161	*MON1B*	-0.3	< .001
hsa-miR-3161	*RPS6KB1*	-0.32	< .001
hsa-miR-3161	*EIF4G2*	-0.24	< .001
hsa-miR-3161	*TRMT5*	-0.3	< .001
hsa-miR-3161	*ZBED3*	-0.27	< .001
hsa-miR-3161	*SLC38A2*	-0.24	< .001
hsa-miR-3161	*SETDB2*	-0.27	< .001
hsa-miR-3161	*ABI2*	-0.28	< .001
hsa-miR-3161	*POU2F1*	-0.27	< .001
hsa-miR-3161	*ANP32E*	-0.25	< .001
hsa-miR-3161	*LBR*	-0.27	< .001
hsa-miR-3161	*MBNL1*	-0.32	< .001
hsa-miR-3161	*SREK1IP1*	-0.24	< .001
hsa-miR-3161	*CBR1*	-0.25	< .001
hsa-miR-3161	*DIP2A*	-0.25	< .001
hsa-miR-3161	*UBE2Q1*	-0.3	< .001
hsa-miR-3161	*SRSF2*	-0.27	< .001
hsa-miR-3161	*ZNF367*	-0.21	< .001
hsa-miR-3161	*NUDT21*	-0.31	< .001
hsa-miR-3161	*CDK1*	-0.19	< .001
hsa-miR-3161	*TOMM20*	-0.26	< .001
hsa-miR-3161	*NR2C2*	-0.24	< .001
hsa-miR-3161	*LRRC57*	-0.23	< .001
hsa-miR-3161	*NGRN*	-0.25	< .001
hsa-miR-3161	*PTAR1*	-0.28	< .001
hsa-miR-3177-5p	*PRRG4*	-0.18	< .001
hsa-miR-3181	*FAM3C*	-0.18	0.008
hsa-miR-3181	*ATP5E*	-0.13	0.061
hsa-miR-3181	*KLHDC3*	-0.17	0.061
hsa-miR-3615	*KHSRP*	-0.17	0.008
hsa-miR-3615	*NIPA2*	-0.25	0.008
hsa-miR-3615	*ZYG11B*	-0.18	0.008
hsa-miR-3615	*HIST1H1B*	-0.09	0.008
hsa-miR-3615	*PPP6R1*	-0.2	0.011
hsa-miR-3615	*WNT4*	-0.2	0.011
hsa-miR-3615	*CCNDBP1*	-0.27	0.011
hsa-miR-3615	*CTC1*	-0.21	0.011
hsa-miR-3615	*VASP*	-0.19	0.013
hsa-miR-3615	*ARF6*	-0.21	0.013
hsa-miR-3615	*LRCH3*	-0.19	0.013
hsa-miR-3615	*ZBTB7A*	-0.21	0.021
hsa-miR-3615	*SRM*	-0.1	0.027
hsa-miR-3615	*OPA3*	-0.21	0.042
hsa-miR-3615	*WDR12*	-0.14	0.042
hsa-miR-3615	*SPRY1*	-0.13	0.047
hsa-miR-3615	*ATP9A*	-0.09	< .001
hsa-miR-3615	*RPS8*	-0.18	< .001
hsa-miR-3615	*ALDOA*	-0.22	< .001
hsa-miR-3615	*COX20*	-0.29	< .001
hsa-miR-3615	*BMPR2*	-0.22	< .001
hsa-miR-378e	*TGFB2*	-0.14	0.052
hsa-miR-4510	*CDH7*	0.25	< .001
hsa-miR-4638-5p	*GINM1*	-0.16	0.073
hsa-miR-4638-5p	*OGFOD1*	-0.15	0.073
hsa-miR-4638-5p	*ALDH2*	-0.17	0.073
hsa-miR-4638-5p	*FEM1A*	-0.19	0.073
hsa-miR-4638-5p	*CNBP*	-0.25	0.073
hsa-miR-4638-5p	*RSBN1L*	-0.17	0.073
hsa-miR-4638-5p	*FAM120AOS*	-0.19	0.073
hsa-miR-4638-5p	*HMGB1*	-0.15	0.073
hsa-miR-4638-5p	*CAPZA2*	-0.14	0.073
hsa-miR-4638-5p	*PEX26*	-0.2	0.073
hsa-miR-4638-5p	*PI4K2B*	-0.18	0.077
hsa-miR-4638-5p	*RAB10*	-0.14	0.077
hsa-miR-4638-5p	*RRP36*	-0.14	0.077
hsa-miR-4638-5p	*OXA1L*	-0.22	0.077
hsa-miR-4638-5p	*ZBTB43*	-0.21	0.077
hsa-miR-4638-5p	*FGFR1OP2*	-0.19	0.079
hsa-miR-4638-5p	*HSD17B12*	-0.14	0.079
hsa-miR-4638-5p	*SGPP2*	-0.22	0.079
hsa-miR-4638-5p	*ERAP2*	-0.24	0.079
hsa-miR-4638-5p	*ZNF652*	-0.14	0.079
hsa-miR-4638-5p	*GABPB1*	-0.19	0.081
hsa-miR-4638-5p	*CIAO1*	-0.1	0.081
hsa-miR-4638-5p	*MTMR10*	-0.15	0.081
hsa-miR-4638-5p	*KCMF1*	-0.12	0.081
hsa-miR-4638-5p	*ZNF485*	-0.17	0.081
hsa-miR-4638-5p	*GNPNAT1*	-0.16	0.087
hsa-miR-4638-5p	*YIPF5*	-0.16	0.087
hsa-miR-4638-5p	*SNRPD1*	-0.21	0.087
hsa-miR-4638-5p	*TSPAN6*	-0.12	0.092
hsa-miR-4638-5p	*ZNF207*	-0.09	0.092
hsa-miR-4638-5p	*MRI1*	-0.13	0.092
hsa-miR-4638-5p	*MAPK1*	-0.15	0.092
hsa-miR-4638-5p	*RBM23*	-0.13	0.092
hsa-miR-4638-5p	*NUP93*	-0.13	0.092
hsa-miR-4638-5p	*RPL34*	-0.11	0.092
hsa-miR-4638-5p	*GNB4*	-0.14	0.092
hsa-miR-4638-5p	*RNF11*	-0.16	0.092
hsa-miR-4638-5p	*RAB9A*	-0.18	0.092
hsa-miR-4638-5p	*IRF1*	-0.18	0.092
hsa-miR-4638-5p	*TEP1*	-0.13	0.092
hsa-miR-4638-5p	*SCO1*	-0.19	0.092
hsa-miR-4638-5p	*CDK4*	-0.09	0.092
hsa-miR-4638-5p	*UGGT1*	-0.13	0.092
hsa-miR-4638-5p	*PRPF4*	-0.17	0.092
hsa-miR-4638-5p	*PNPT1*	-0.11	0.092
hsa-miR-4638-5p	*ENSA*	-0.11	0.092
hsa-miR-4638-5p	*CNKSR3*	-0.14	0.092
hsa-miR-4638-5p	*FOXK1*	-0.09	0.092
hsa-miR-4638-5p	*TRMT10B*	-0.18	0.092
hsa-miR-4638-5p	*MELK*	-0.16	0.092
hsa-miR-4638-5p	*ZNF561*	-0.16	0.092
hsa-miR-4638-5p	*KIAA1919*	-0.13	0.092
hsa-miR-4638-5p	*CCS*	-0.14	0.092
hsa-miR-4638-5p	*GK5*	-0.13	0.092
hsa-miR-4638-5p	*ZNF682*	-0.18	0.092
hsa-miR-4638-5p	*SF3B1*	-0.08	0.096
hsa-miR-4638-5p	*TNFAIP8L1*	-0.12	0.096
hsa-miR-4638-5p	*PSMB5*	-0.14	0.097
hsa-miR-4638-5p	*MCM4*	-0.12	0.097
hsa-miR-4638-5p	*SMG1*	-0.1	0.097
hsa-miR-4638-5p	*LRIG2*	-0.13	0.097
hsa-miR-4638-5p	*OPTN*	-0.22	< .001
hsa-miR-4638-5p	*ZNF655*	-0.17	< .001
hsa-miR-4660	*CTBS*	-0.2	< .001
hsa-miR-4731-3p	*HSP90AB1*	-0.16	0.015
hsa-miR-4731-3p	*IKZF4*	-0.22	0.015
hsa-miR-4731-3p	*SPTLC2*	-0.24	0.023
hsa-miR-4731-3p	*N4BP1*	-0.24	0.023
hsa-miR-4731-3p	*NRF1*	-0.25	0.023
hsa-miR-4731-3p	*SLC38A2*	-0.21	0.023
hsa-miR-4731-3p	*STAT5A*	-0.2	0.027
hsa-miR-4731-3p	*CHEK1*	-0.22	0.027
hsa-miR-4731-3p	*MFSD6*	-0.34	0.027
hsa-miR-4731-3p	*PPP2R5E*	-0.25	0.027
hsa-miR-4731-3p	*TMED10*	-0.24	0.033
hsa-miR-4731-3p	*ZNF678*	-0.27	0.033
hsa-miR-4731-3p	*HFE*	-0.22	0.034
hsa-miR-4731-3p	*TJP1*	-0.25	0.034
hsa-miR-4731-3p	*TFCP2*	-0.17	0.034
hsa-miR-4731-3p	*ZIC5*	-0.2	0.034
hsa-miR-4731-3p	*MYO10*	-0.16	0.034
hsa-miR-4731-3p	*ASB6*	-0.21	0.034
hsa-miR-4731-3p	*PIP4K2A*	-0.17	0.034
hsa-miR-4731-3p	*SRSF2*	-0.21	0.034
hsa-miR-4731-3p	*ZBTB40*	-0.21	0.034
hsa-miR-4731-3p	*TPM2*	-0.24	0.034
hsa-miR-4731-3p	*SRSF1*	-0.19	0.038
hsa-miR-4731-3p	*PTPN3*	-0.21	0.041
hsa-miR-4731-3p	*ANKS6*	-0.22	0.041
hsa-miR-4731-3p	*PTMA*	-0.17	0.055
hsa-miR-4731-3p	*SREK1*	-0.19	0.058
hsa-miR-4731-3p	*TRAM1*	-0.17	0.059
hsa-miR-4731-3p	*GALNT7*	-0.18	0.059
hsa-miR-4731-3p	*QKI*	-0.16	0.059
hsa-miR-4731-3p	*SKIL*	-0.17	0.059
hsa-miR-4731-3p	*GATAD2B*	-0.22	0.059
hsa-miR-4731-3p	*ANKRD40*	-0.16	0.059
hsa-miR-4731-3p	*TAF1D*	-0.13	0.059
hsa-miR-4731-3p	*VCPIP1*	-0.19	0.059
hsa-miR-4731-3p	*CCDC43*	-0.17	0.059
hsa-miR-4731-3p	*DIDO1*	-0.17	0.06
hsa-miR-4731-3p	*KCNC4*	-0.18	0.06
hsa-miR-4731-3p	*MAP3K9*	-0.18	0.068
hsa-miR-4731-3p	*EIF5A2*	-0.19	0.068
hsa-miR-4731-3p	*ATP9A*	-0.13	0.074
hsa-miR-4731-3p	*SHOC2*	-0.21	0.074
hsa-miR-4731-3p	*SLC1A4*	-0.12	0.074
hsa-miR-4731-3p	*GAN*	-0.16	0.074
hsa-miR-4731-3p	*SFXN5*	-0.17	0.074
hsa-miR-4731-3p	*ZNF432*	-0.15	0.074
hsa-miR-4731-3p	*ZNF585A*	-0.1	0.076
hsa-miR-4731-3p	*ACBD5*	-0.3	< .001
hsa-miR-4731-3p	*SOS1*	-0.28	< .001
hsa-miR-4731-3p	*FBXL5*	-0.26	< .001
hsa-miR-4731-3p	*NDUFA5*	-0.31	< .001
hsa-miR-4731-3p	*CLSTN3*	-0.24	< .001
hsa-miR-4731-3p	*THYN1*	-0.26	< .001
hsa-miR-4731-3p	*RABGEF1*	-0.26	< .001
hsa-miR-4731-3p	*MYO6*	-0.33	< .001
hsa-miR-4731-3p	*TLK1*	-0.25	< .001
hsa-miR-4731-3p	*LYN*	-0.24	< .001
hsa-miR-500a-3p	*C18orf25*	-0.24	< .001
hsa-miR-520e	*CDIPT*	-0.29	0.075
hsa-miR-520e	*WWC1*	-0.23	0.075
hsa-miR-520e	*PDPK1*	-0.3	0.075
hsa-miR-520e	*ZNF12*	-0.37	0.075
hsa-miR-520e	*KBTBD6*	-0.29	0.075
hsa-miR-520e	*ZBTB33*	-0.23	0.075
hsa-miR-520e	*TSPAN6*	-0.24	0.094
hsa-miR-520e	*TFAP4*	-0.22	0.094
hsa-miR-520e	*SUGP1*	-0.3	0.094
hsa-miR-520e	*SERINC1*	-0.22	0.094
hsa-miR-520e	*NUDT3*	-0.29	0.094
hsa-miR-520e	*KCNJ8*	-0.17	0.094
hsa-miR-520e	*ZNFX1*	-0.29	0.094
hsa-miR-520e	*ABI2*	-0.33	0.094
hsa-miR-520e	*EFCAB11*	-0.22	0.094
hsa-miR-520e	*TM4SF5*	-0.28	0.094
hsa-miR-520e	*ZNF7*	-0.24	0.094
hsa-miR-520e	*HSPA13*	-0.25	0.094
hsa-miR-520e	*NEK8*	-0.27	0.094
hsa-miR-520e	*ITGA2*	-0.29	0.094
hsa-miR-520e	*FOXK1*	-0.27	0.094
hsa-miR-520e	*CYB5A*	-0.23	0.094
hsa-miR-520e	*BLCAP*	-0.34	0.094
hsa-miR-520e	*BSCL2*	-0.26	0.094
hsa-miR-520e	*KBTBD2*	-0.35	0.094
hsa-miR-520e	*ZMAT3*	-0.17	0.094
hsa-miR-520e	*ARL10*	-0.23	0.094
hsa-miR-520e	*CAPZA2*	-0.31	0.094
hsa-miR-520e	*ZNF805*	-0.28	0.094
hsa-miR-520e	*SLC35F6*	-0.26	0.094
hsa-miR-520e	*ZNF174*	-0.37	< .001
hsa-miR-520e	*SLC6A4*	-0.36	< .001
hsa-miR-520e	*BMP8B*	-0.28	< .001
hsa-miR-520e	*ZNF426*	-0.31	< .001
hsa-miR-520e	*ABHD15*	-0.35	< .001
hsa-miR-525-5p	*KCNH1*	0.21	< .001
hsa-miR-583	*NKD1*	0.08	0.068
hsa-miR-583	*SIGLEC9*	-0.25	< .001
hsa-miR-583	*KIF2C*	0.2	< .001
hsa-miR-583	*PDZRN4*	-0.25	< .001

**Fig 1:**
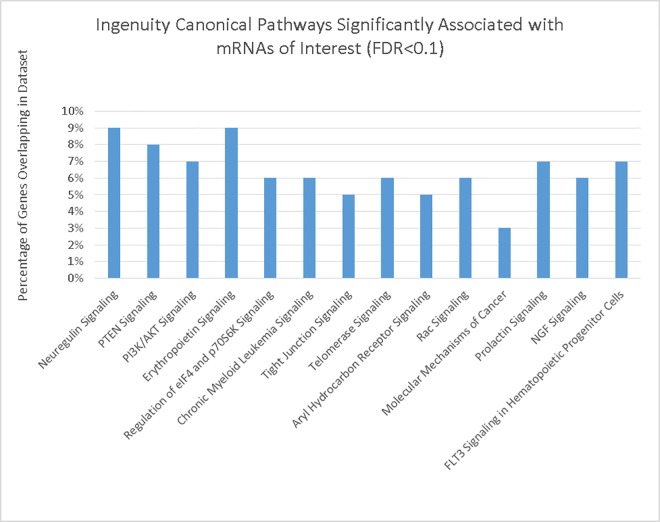
IPA canonical pathways associated with 270 genes targeted by miRNAs associated with *TERT*.

Using IPA to construct pathways enriched with the 270 genes with an FDR of <0.1, we identified 14 pathways (Fig 1). Although less than 10% of genes in each pathway were included in our gene list, these pathways were significantly enriched by our genes. The enriched pathways included: neuregulin signaling, PTEN signaling, PI3K/AKT signaling, erythropoietin signaling, regulation of EIF4 and p706SK signaling, chronic myeloid leukemia signaling, tight junction signaling, telomerase signaling, aryl hydrocarbon receptor signaling, Rac signaling, molecular mechanisms of cancer, prolactin signaling NGF signaling, and FLT3 signaling in hematopoietic progenitor cells.

## Discussion

These data suggest a direct association between miRNAs and TL as well as between *TERT* rs2736118 and miRNA differential expression between carcinoma tissue and normal colonic mucosa. While TERT expression is low in most somatic cells, endothelial cells represent an established exception and associations observed for differential expression between carcinoma and normal mucosa is a likely extension of that observation. Our results add support to the hypothesis that miRNAs are associated with TL and that genetic variation in telomere-related genes influence miRNA expression levels. Given the number of miRNAs altered by *TERT* rs2736118, our ability to show associations between altered miRNAs and their targeted mRNA, and the pathways represented by these target genes, we believe that our data support the hypothesis that *TERT* has functions beyond that of regulating TL. Focusing only on those miRNAs that were statistically associated with target mRNA expression in colon tissue, we identified 14 canonical pathways that were associated significantly with these *TERT*-regulated miRNAs after adjustment for multiple comparison, 13 of which were not associated with telomerase.

There have been few reports of miRNAs associated with TL that have looked at actual miRNA expression levels in conjunction with TL; our sample size is by far the largest study to date to examine miRNA expression with TL. One study with only two samples compared shortened telomeres to intact telomeres and showed that 47 miRNAs had over a twofold difference in expression levels [27]. MiR-155, miR-138, and miR-34a,b,c also have been hypothesized as being associated with TL [3, 5]; none of these miRNAs were associated in a linear fashion with TL in our study. The miRNAs associated with TL in our data for the most part increased TL with increased miRNA expression. Differences in association between our study and others may in part be from the methods of analysis. We assessed a linear association, while other studies have often used categorical variables and looked at either short or long TL, which could account for some of the differences observed.

A limitation of the study is that we are unable to confirm our observed associations between TL and miRNAs in bio-functional studies; we encourage others to do so. However, support for our findings come from several sources. First, we have previously shown that 26 of the 34 miRNAs associated with TL were differentially expressed between colorectal carcinoma and normal colonic mucosa and 28 of the 34 were dysregulated between carcinoma and adenomatous polyp tissue; three miRNAs were dysregulated between normal colonic mucosa and adenomatous polyp tissue [18]. Interestingly, the only miRNA that was directly associated with TL, miR-487b, was the only miRNA that was not associated with dysregulation between carcinoma, adenoma, and/or normal mucosa. Additionally, using bioinformatics tools to identify the target genes for the dysregulated miRNAs associated with TL, we observed that 12 of these miRNAs associated with TL target genes (summary in Table 5) are components of the alternative lengthening of telomeres pathway (ALT) and Shelterin [28]. Shelterin is a conserved protein component on telomeres that serves as a functional framework of telomere chromatin pathways. ALT is a telomerase-independent pathway of lengthening telomeres in human cells. Given that there is no overlap between TERT SNP-associated miRNAs and TL-associated miRNAs, and that a large proportion of these miRNAs target genes in the ALT pathway, it is possible that miRNAs regulate TL through this alternative mechanism. As TL has been shown to be associated with CRC, the associations between TL and miRNA expression may unique clinical significance.

**Table 5:** Summary of miRNAs related to telomere length and *TERT* rs2736118 differentially expressed in colorectal cancer.

	Differentially Expressed:			
miRNA	T—N^1^	T—AD	AD—N	ALT Gene(s) Targeted
Telomere Length				
hsa-miR-1185-2-3p		x		
hsa-miR-1207-5p	x	x		*CBX5*, *TERT*, *TP53*
hsa-miR-1226-5p	x	x		*CBX5*, *RAD18*
hsa-miR-1229-5p	x	x		* *
hsa-miR-1247-3p			x	*FANCA*, *RAD51L3-RFFL*, *SMC5*
hsa-miR-134	x	x		
hsa-miR-3141			x	
hsa-miR-3158-5p	x	x		
hsa-miR-3162-5p	x	x		
hsa-miR-3194-5p	x	x		
hsa-miR-3196	x	x		
hsa-miR-3200-5p	x	x		
hsa-miR-3937				*FANCA*, *CBX5*
hsa-miR-4253		x		* *
hsa-miR-4459	x	x		*TERF2*, *ERCC1*, *HSP90B1*, *SMC5*
hsa-miR-4496	x			*RAD50*, *RAD51B*, *RAP1B*
hsa-miR-4497	x	x		*FANCA*
hsa-miR-4530	x	x		* *
hsa-miR-4535	x	x		* *
hsa-miR-4673	x	x		*XRCC3*
hsa-miR-4689				*CDKN1A*, *RAD18*, *RAD50*, *XRCC3*
hsa-miR-4739	x	x		
hsa-miR-487b				
hsa-miR-5006-5p	x	x		
hsa-miR-5088	x	x		
hsa-miR-5195-3p	x	x		* *
hsa-miR-575	x	x	x	*RAD51*
hsa-miR-601	x	x		* *
hsa-miR-6076	x	x		* *
hsa-miR-662	x	x		* *
hsa-miR-718	x	x		* *
hsa-miR-762	x	x		*CDK2*, *RAD54L2*
hsa-miR-887	x	x		* *
hsa-miR-939-5p	x	x		*CDKN1A*
*TERT* rs2736118				
hsa-miR-1203	x	x		
hsa-miR-1237-5p	x			
hsa-miR-125b-1-3p				
hsa-miR-1266				
hsa-miR-1276				
hsa-miR-1295b-3p				*WRN*
hsa-miR-1323				* *
hsa-miR-1470	x			* *
hsa-miR-184				* *
hsa-miR-206	x	x		*HSP90B1*
hsa-miR-302c-5p				*CBX4*
hsa-miR-3122				*PARP2*
hsa-miR-3131				* *
hsa-miR-3150b-3p				*CDK2*, *CDKN1A*, *RAD18*, *RAD50*, *XRCC3*
hsa-miR-3161	x			* *
hsa-miR-3177-5p	x			* *
hsa-miR-3181	x			* *
hsa-miR-3186-3p				* *
hsa-miR-3189-5p				* *
hsa-miR-339-3p				* *
hsa-miR-34c-3p				* *
hsa-miR-3615	x			* *
hsa-miR-3660				* *
hsa-miR-3679-3p		x	x	* *
hsa-miR-3680-3p				*CBX4*
hsa-miR-378e				*RAP1B*
hsa-miR-3922-5p				* *
hsa-miR-4300				* *
hsa-miR-4303				* *
hsa-miR-4421	x			* *
hsa-miR-4436a				* *
hsa-miR-4444				* *
hsa-miR-4450				* *
hsa-miR-4461				* *
hsa-miR-4479		x		* *
hsa-miR-4489				* *
hsa-miR-4510	x	x		*TP53*, *MRE11A*
hsa-miR-4519				* *
hsa-miR-4522	x			*PARP2*
hsa-miR-4526				* *
hsa-miR-4638-5p				* *
hsa-miR-4654				* *
hsa-miR-4657	x		x	* *
hsa-miR-4659b-3p				*CDKN1A*
hsa-miR-4660				
hsa-miR-4674		x		
hsa-miR-4676-5p				*RAD51*
hsa-miR-4682				*CDKN1A*, *TP53*
hsa-miR-4684-3p				*NR2F2*
hsa-miR-4715-5p				*CBX4*
hsa-miR-4717-3p				*WRN*
hsa-miR-4731-3p				* *
hsa-miR-4732-5p	x		x	* *
hsa-miR-4748				*WRN*
hsa-miR-500a-3p				* *
hsa-miR-516b-5p				* *
hsa-miR-518c-5p				* *
hsa-miR-5195-5p				* *
hsa-miR-519e-5p				* *
hsa-miR-520e	x		x	*CBX5*, *FANCA*, *NR2F2*
hsa-miR-525-5p				* *
hsa-miR-550a-5p	x			*RAD51*
hsa-miR-551b-5p				*ERCC1*
hsa-miR-5572				* *
hsa-miR-5584-5p				*CDKN1A*, *RAD50*, *RAD54L2*
hsa-miR-566				*CBX5*
hsa-miR-583	x		x	*CBX5*, *CDKN1A*
hsa-miR-616-3p	x		x	* *
hsa-miR-639		x		*CDKN1A*
hsa-miR-6509-5p				* *
hsa-miR-658	x			*HSP90B1*, *SMC5*
hsa-miR-659-3p				* *
hsa-miR-6716-5p				*CBX5*
hsa-miR-6718-5p		x	x	* *
hsa-miR-708-5p		x		*TOP2A*

^1^’T’ signifies ‘Tumor tissue’, ‘N’ signifies ‘Normal colonic mucosa’, and ‘AD’ signifies ‘Adenoma tissue’.

We also observed that genetic variation in *TERT*, which we have previously reported as being associated with TL and cancer [6, 14], was associated with miRNA expression levels. Many of these miRNAs have been shown to be dysregulated in CRC [18] and 26 of these miRNAs target ALT pathway genes (Table 5). One of the pathways enriched by genes targeted by dysregulated miRNAs is ‘telomerase signaling’. Since telomerase is generally not expressed in non-tumor somatic tissue but is expressed in the majority of tumors, it is logical that *TERT* rs2836118 was not associated with miRNA expression in normal colonic mucosa, but only in differential miRNA expression between carcinoma and normal colorectal mucosa. Telomerase is often reactivated by TERT upregulation, which gives tumor cells their immortal quality, and this is thought to be a key step in the adenoma-carcinoma transition in human tumorigenesis [29]. As stated previously, we identified miRNAs that were differentially expressed between normal colonic mucosa and adenomatous polyp tissues as well as between adenoma and carcinoma tissues, which show the changes in miRNA expression as tissue progresses from normal to adenomatous to cancerous [18]. Both hsa-miR-520e and hsa-miR-583 were differentially expressed between normal colonic mucosa and adenomatous polyp tissue [18]; in this study we show that they also regulate mRNA expression. These two miRNAs regulated 40 genes, including *ITGA2*, *BMP8B*, and *PDPK1*, which were found in numerous pathways in IPA, including PTEN Signaling, BMP signaling pathway, and Basal Cell Carcinoma Signaling. Hsa-miR-520e was associated with *CBX5*, *FANCA* and *NR2F2*, and hsa-miR-583 was associated with *CBX5* and *CDKN1A*, all of which are part of the ALT pathway. We also identified six miRNAs that were downregulated with the AG/GG (heterozygous-homozygous recessive) genotype of rs2736118 that we previously found were dysregulated between adenomatous and carcinoma tissue; these miRNAs were: hsa-miR-206, hsa-miR-4510, hsa-miR-4674, hsa-miR-639, hsa-miR-6718-5p, and hsa-miR-708-5p. This supports the hypothesis that TERT regulates miRNA expression and this influences the formation of adenomatous tissue and contributes to tumorigenesis.

The association of *TERT* rs2736118 genotype with miRNA expression levels may provide additional insight into pathways in which *TERT* is involved. We therefore examined genes and pathways regulated by the miRNAs that were influenced by *TERT* rs2736118. We observed that genetic variation in *TERT* was associated with dysregulation of 75 miRNAs, which in turn could influence translation and function of over 6000 genes. While many genes are regulated by these miRNAs, it is not clear which of these are related to colorectal cancer. Evaluation of over 6000 genes leads to a very non-specific assessment of potential functionality. To better focus on functionality we attempted to cluster genes together into what could be potentially a more common function and we also assessed seed regions. Using a cluster analysis approach, we still had over fifty pathways that also lacked specificity to colorectal cancer. Focusing on seed regions, we were left with 73 seed regions and did not minimize the potential number of genes that could be associated with *TERT*. Thus, we took an approach that focused only on those genes where the gene targeted by the miRNA was linearly associated with the corresponding mRNA expression in colorectal cancer. This reduced the number of genes and pathways considerably and was specific to colorectal cancer. However, while we gained specificity, we undoubtedly were limited in the target genes we assessed. We believe that the pathway information obtained is meaningful, but most certainly not complete. These data do however support the hypothesis that TERT has non-TL-related pathways [29, 30] that may be acting through altered miRNA expression.

In addition to its established role in telomere maintenance, it has been proposed that TERT operates through other pathways. Further examples of TERT involvement in non-TL related pathways include both NFκB complex and the Wnt/β-catenin pathways, which have been hypothesized as being telomerase-targeted pathways as both are pathways related to inflammation [5]; these pathways were both represented by our target genes in IPA although they are not shown in Fig 1. Most notably, the NFκB-signaling pathway activation has been suggested as being related to TERT [31]. TERT levels also have been associated with differential expression of genes involved in development/morphogenesis, signal transduction, and cell adhesion-signaling pathways [32, 33]. Our data also suggest that associations between *TERT* and colorectal cancer, possibly mediated by miRNAs, involve numerous pathways other than telomerase. These pathways involve tumor suppressor genes (such as PTEN signaling), apoptosis (PI3K/AKT signaling, NGF signaling, and FLT3 signaling), angiogenesis (Erythropoietin signaling), immune regulation and response (Prolactin signaling and Aryl Hydrocarbon Receptor), cell migration and proliferation (Neuregulin signaling), and growth factor activity (NGF signaling and EIF4 and p70S6K signaling). Another enriched pathway, Tight Junction Signaling, is important in cellular communication and facilities crosstalk across several of these signaling pathways including PI3K/AKT and PTEN [34].

Additionally, specific genes such as *STAT5A*, *MELK*, *MAPK1* and *MAP3K9*, *RELA* (part of the NFKB1 complex), *CDK1*, tumor necrosis factor genes, *IRF1*, and TGFβ-related genes were associated with miRNAs differentially expressed by *TERT* genotype. A number of translocations associated with cancer involve the Ig heavy chain locus on chromosome 14. For example t(8;14) brings c-MYC from chromosome 8 to the IgH gene locus causing an overexpression of c-MYC in Burkitt lymphoma. A t(18;14) translocation moving Bcl-2 from chromosome 18 to the IgH locus on 14 is involved in follicular lymphoma. Also CCND1 is involved in a t(11;14) translocation bringing cyclin d1 to the IgH resulting in cell cycle dysregulation in Mantle cell lymphoma. Studies suggest that these cancers are associated with telomere length [35–38] as well as with miRNA levels [39]. We show that *TERT* regulation of miRNAs alters Bcl-2 and an enriched pathway associated with the dysregulated genes is chronic myeloid leukemia, which has overlapping genes with some of the lymphomas previously studied. Our data support the hypothesis that TERT is involved in disease processes that include non-TL-related pathways.

One of the major strengths of our study was the availability of miRNA expression data, SNP data, and mRNA data on a large population. We have previously analyzed these SNPs with both TL and colon cancer, which helps facilitate the interpretation of our data. Additionally, we applied a FDR to guide our interpretation of meaningful results. Larger samples would have enabled us to split the sample and replicate our findings with a second data set. While we are unable to replicate these findings in a second data set, we encourage others with similar data to undertake such analysis. There are many available tools to assist in identifying verified mRNA targets for miRNA. In this study we used several bioinformatics tools to better understand function of these miRNAs, however all bioinformatics tools have limitations as to the extent of the knowledge and the specificity of the information to specific miRNAs. To have a more focused assessment of pathways, we restricted our bioinformatics assessment to only those targeted genes significantly associated with miRNA. Although functionality data of specific miRNA and TERT associations are not available or feasible from data available to us, we believe that the information provided gives direction for more laboratory-based studies that can directly test these associations.

## Conclusions

In conclusion, our data suggest that miRNAs are involved in regulating TL. *TERT* appears to influence carcinoma/normal mucosa differential expression. Given the number of miRNAs associated with TL, *TERT* rs2736118 and ALT genes, our data also support the hypothesis that telomere-related genes, in addition to affecting TL, impact non-TL-related functions that can importantly influence cancer risk.

## Abbreviations

CRC: Colorectal cancer
FDR: False discovery rate
IPA: Ingenuity pathway analysis
FPMRP: Kaiser Permanente Medical Research Program
mRNA: messenger RNA
miRNA: microRNA
PTEN: Phosphate and tensin homolog
PI3K: Phosphatidylinositide 3-kinases
AKT: Protein kinase B
QC: Quality Control
qPCR: Quantitative polymerase chain reaction
RNA-seq: RNA sequencing
SNP: Single nucleotide polymorphism
TERT: Telomerase reverse transcriptase
TL: Telomere length
T/S ratio: Telomere-to-single copy gene
